# A Short Manual of Analytical Chemistry, Qualitative and Quantitative—Inorganic and Organic

**Published:** 1892-04

**Authors:** 


### Bibliography.

A Short Manual of Analytical Chemistry, Qualitative and Quantitative—Inorganic and Organic. By John Muter, M.A., Ph.D., etc., Analyst to the Metropolitan Asylums Board, etc. Edited by Claude C. Hamilton, M.D., Ph.G., Professor of Analytical Chemistry in the University Medical College, Kansas City. Philadelphia: P. Blakiston, Son & Co., 1891.

The reviser of this book states in his preface "that it has been his effort to let the original text remain as little altered as circumstances would admit." It is unfortunate that the reviser adopted this course, for perhaps there is no book on science in the English language which has passed through four editions and still contains so many inaccuracies and such clumsily-worded sentences as this book of Muter's. Here and there throughout the book abbreviations are used which certainly must be puzzling to the student. For instance, on page 62 the abbreviation HAc is used. Few beginners in qualitative analysis would understand that it signified acetic acid, for in the special tests for that acid on page 47 no reference is made to such an abbreviation. Again, on page 69, few students would understand that S.V.R. signified ordinary alcohol (spiritus vini rectificatus).

On page 12, under Lead, it is stated that in practising upon a solution of Pb $(C_2H_3O_2)_2$, "$NH_4HO$ causes a *white* precipitate of a *white* basic nitrate—Pb $(NO_3HO)$." It would be interesting to know the author's explanation of the production of a nitrate from an acetate and a hydrate. Such statements are inexplicable in a book that has passed through four editions, and has finally been revised and now appears practically in its fifth edition.

Notwithstanding these blemishes, the book possesses much that is good. The table for the detection of the metal in a solution containing one base only is an admirable one. The tables for the detection of metals in complex mixtures, barring the obscurity in statement and brevity in description, which latter, of course, is almost a necessity in the construction of such tables, are good.

The scheme for the detection of acids is one of the best features of the book.

The other chapters on organic analysis, water analysis, etc., are in the main good, with the exception of the chapter on urine analysis, which is sufficiently brief to render it almost useless. The plates accompanying this chapter, in which the microscopic appearances of urinary sediments are pictured, are ludicrous in their atrociousness. B.

